# A Review of Quantitative Instruments for Understanding Breastfeeding Dynamics

**DOI:** 10.1002/gch2.202100019

**Published:** 2021-07-20

**Authors:** Matin Torabinia, Steven D. Rosenblatt, Bobak Mosadegh

**Affiliations:** ^1^ Dalio Institute of Cardiovascular Imaging NewYork‐Presbyterian Hospital and Weill Cornell Medicine New York NY 10021 USA; ^2^ Department of Radiology Weill Cornell Medicine New York NY 10021 USA; ^3^ Department of Otolaryngology‐Head and Neck Surgery Weill Cornell Medicine New York NY 10021 USA

**Keywords:** ankyloglossia, breastfeeding dynamics, breastfeeding instruments, infant latching and feeding, milk removal

## Abstract

Breastfeeding, as a unique behavior of the postpartum period and an ideal source of nourishment, is profoundly impacted by the physiology and behavior of both mothers and infants. For more than three‐quarters of a century, there has been an ongoing advancement of instruments that permit insight into the complex process of latching during breastfeeding, which includes coordinating sucking, swallowing, and breathing. Despite the available methodologies for understanding latching dynamics, there continues to be a large void in the understanding of infant latching and feeding. The causes for many breastfeeding difficulties remain unclear, and until a clearer understanding of the mechanics involved is achieved, the struggle will continue in the attempts to aid infants and mothers who struggle to breastfeed. In this review, the history of development for the most prominent tools employed to analyze breastfeeding dynamics is presented. Additionally, the importance of the most advanced instruments and systems used to understand latching dynamics is highlighted and how medical practitioners utilize them is reported. Finally, a controversial argument amongst pediatric otolaryngolo gists concerning breastfeeding difficulties is reviewed and the urgent need for quantification of latching dynamics in conjunction with milk removal rate through prospective controlled studies is discussed.

## Graphical abstract

The history of development for the most prominent tools employed to analyze breastfeeding dynamics is presented. The importance of the most advanced instruments and systems used by medical practitioners to understand latching is highlighted. Lastly, a controversial argument among pediatric otolaryngologists concerning breastfeeding difficulties is reviewed and the urgent need for quantification of latching dynamics is discussed.

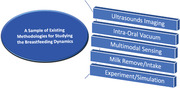

## 1 Background

The postpartum period is a critical time for both mothers and infants, so much so that their interactions influence the physiology and behavior of each other.^[^ ^1^ ^]^ This relationship stresses the importance of the breastfeeding dyad and can be perceived as an inherent and essential characteristic of mammals to support the survival of their offspring. Breastfeeding, as a unique behavior of the postpartum period, and an ideal source of nourishment, increases skin‐to‐skin contact and contributes to more rapid metabolic adaptation in newborns.^[^ ^2^ ^]^ More than two decades ago, breastfeeding was recognized as the most effective international preventive health intervention, with the potential to prevent 13% of deaths among children younger than 5 years of age worldwide.^[^ ^3^ ^]^ Studies have shown that infants who do not receive human milk are at higher risk for a number of medical issues, including necrotizing enterocolitis,^[^ ^4^, ^5^ ^]^ gastrointestinal and upper respiratory tract illness,^[^ ^6^, ^7^ ^]^ and urinary tract infections.^[^ ^8^ ^]^

The World Health Organization suggests that all newborns should consume solely human milk during the first six months of life and continue with proper complementary foods until 2 years of age.^[^ ^9^ ^]^ Breastfeeding has also been recognized as the ideal source of nourishment and the preferred primary feeding modality for infants by the American Association of Pediatrics. In addition, the US Surgeon General published a “Breastfeeding Call to Action” in 2011, touting the benefits of breastfeeding and encouraging all US families to pursue breastfeeding.^[^ ^10^ ^]^ This renewed interest in breastfeeding fueled ongoing clinical and industrial investigations addressing different aspects of breastfeeding, including nutrition quality, breastfeeding duration, positioning, sucking and swallowing frequency, and latching effectiveness.^[^ ^11^, ^12^ ^]^ Throughout this review, we use the term “sucking,” which refers to a complex interaction and coordination of an infant's jaw, hyoid bone, palate, pharynx, and tongue to coordinate milk removal during breastfeeding.^[^ ^13^, ^14^ ^]^ Additionally, the term “sucking microstructure” will be noted in different sections. Sucking microstructure is defined as sets of metrics that comprehensively capture infants’ effort to create a sealed latch onto the mother's nipple and regulate feeding, including the number of sucks, sucks per burst, number of bursts, intra suck interval, and maximal sucking pressure.

The emergence of instruments for the evaluation of breastfeeding began in the 1950s and 1960s, following the development of validated tools designed to evaluate neonatal behavior and health. Although these initial behavioral assessment tools were not developed explicitly to evaluate breastfeeding, they formed a foundation for breastfeeding instruments that appeared later on. Revolutionary measures, like the Apgar score,^[^ ^15^, ^16^ ^]^ exemplified that accurate and precise neonatal behavioral evaluations were possible and could be used to determine outcomes. Subsequent evaluative tools put forth by Graham et al.^[^ ^17^ ^]^ and Scanlon et al.^[^ ^18^ ^]^ presented new tools to score infant outcomes following trauma or exposure to maternal epidural anesthesia, respectively.

Ultimately, Brazelton et al. introduced the Brazelton neonatal behavioral assessment scale (BNBAS), comprising five characteristics that clustered the behavioral patterns of newborns.^[^ ^19^ ^]^ The BNBAS has been employed since the 1970s to guide clinicians and parents on the individual developmental needs of newborns. For example, feeding reflexes were evaluated by an examiner placing a finger along the infant's cheek and mouth to elicit the rooting and sucking reflexes. Although the BNBAS took initial feeding reflexes into consideration, it did not explicitly analyze the complex process of breastfeeding or evaluate for effective feeding from the breast.

The lack of knowledge regarding the complex process of latching engendered the utilization of radiographic tools to analyze rooting and sucking behaviors. Ardran et al. used cineradiographic films of 41 breastfeeding mothers to elucidate the physiology of sucking and swallowing of infants. This study revealed key information regarding nipple position within an infant's oral cavity and the cycle of jaw and tongue movement during feeding.^[^ ^20^ ^]^ Shortly thereafter, mechanical devices, like the suckometer, were developed to record objective measurements of oral pressure changes.^[^ ^21^ ^]^ However, these mechanical devices were limited to bottle feeding and could not be used to evaluate breastfeeding. Consequently, ultrasound imaging (US) became a prominent methodology and provided explicit real‐time descriptions of feeding movements. US allowed a non‐invasive and radiation‐free tool to obtain real‐time information about infant latching and feeding movements. US studies by Smith et al.,^[^ ^22^ ^]^ Selley et al.,^[^ ^23^ ^]^ and Weber et al.^[^ ^24^ ^]^ determined the anatomy of the infant's mouth during breastfeeding. In addition, other research groups used video recordings to characterize the behaviors of the infant during latching,^[^ ^25^ ^]^ and to determine milk flow and sucking rates throughout breastfeeding.^[^ ^26^ ^]^

**Figure 1:**
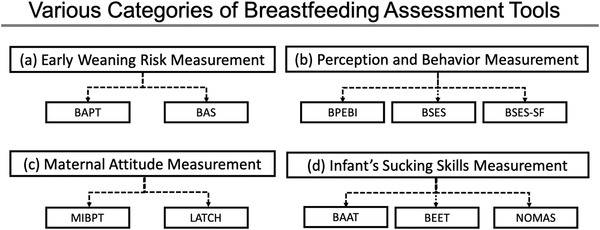
Illustration of various breastfeeding assessment tools. a) Breastfeeding attrition prediction tool (BAPT),^[^ ^31^ ^]^ breastfeeding assessment score (BAS).^[^ ^32^ ^]^ b) Breastfeeding personal efficacy beliefs inventory (BPEBI),^[^ ^33^ ^]^ breastfeeding self‐efficacy scale (BSES),^[^ ^34^, ^35^ ^]^ breastfeeding self‐efficacy scale—short form (BSES‐SF).^[^ ^36^ ^]^ c) Mother–infant breastfeeding progress tool (MIBPT),^[^ ^37^ ^]^ LATCH scoring system (LATCH).^[^ ^38^ ^]^ d) Bristol breastfeeding assessment tool (BAAT),^[^ ^39^ ^]^ breastfeeding evaluation an education tool (BEET),^[^ ^40^ ^]^ neonatal oral‐motor assessment scale (NOMAS).^[^ ^41^ ^]^

From the 1970s‐1990s, US emerged as the preferred modality to measure several parameters of sucking microstructure. Through US, one could identify oral structures, visualize feeding movements, and develop a clinical algorithm for swallow detection and sucking episodes. Additionally, US is safe, relatively low cost, and does not subject infants to ionizing radiation. However, US does not measure oral pressure changes and its use is limited to those with access to appropriate hardware and training. Additionally, differences in imaging techniques can often produce different results which can lead to varied interpretations of the imaging output. These limitations led to further advancement of alternative latching instruments and other experimental and computational efforts in this field. These aforementioned techniques brought two primary theories of infant milk extraction into the spotlight: sucking versus mouthing.^[^ ^27^, ^28^, ^29^, ^30^ ^]^ For the sucking theory, scientists argue that milk extraction relies primarily on the development of subatmospheric pressures within the oral cavity. For mouthing, which is defined as squeezing contents out of the nipple by compression between the jaws or other mouthparts, it is thought that coordinated squeezing of the nipple‐areola complex from the mouth and tongue drives milk flow. In this review, we present some of the remarkable efforts that have taken place in recent decades to objectively assess infant feeding. **Figure** **1** shows various breastfeeding assessment instruments where they fall into four different categories, early weaning risk measurement, perception and behavior measurement, maternal attitude measurement, and infant's sucking skills measurement.

## 2 Methodology

The current and most widely used methodologies for breastfeeding assessment include US, intra‐oral vacuum, multimodal sensing (the synchronization of two or more measurement modalities assessing feeding movements), milk removal/intake, and computational modeling, shown in **Table** **1**. The following sections summarize the existing and most widely used applications of these methodologies in the assessment of breastfeeding.

**Table 1:** Summarizes sample of existing methodologies for studying the breastfeeding dynamics

Methods	Examples
Ultrasounds Imaging	Tracking Infant's mouth and nipple boundary movement^[^ ^42^ ^]^
	Depicting the tongue and the palate via Submental US image^[^ ^43^ ^]^
	Monitoring the movement and position of the tongue during both breast‐ and bottle‐feeding^[^ ^44^ ^]^
Intra‐Oral Vacuum	Vacuum measurement of the intra‐oral cavity during breastfeeding^[^ ^45^ ^]^
	A breastfeeding diagnostic device to measure sucking microstructure^[^ ^46^ ^]^
	Device with built‐in force sensors^[^ ^47^ ^]^
Multimodal Sensing	Synchronization of the endocavity US probe in conjunction with recorded intra‐oral vacuum^[^ ^48^ ^]^
	Placement of a US transducer during bottle‐feeding together with an intra‐oral vacuum measurement using a catheter situated adjacent to the mother's nipple^[^ ^49^ ^]^
	The NTrainer system incorporating a traditional pacifier and a computer‐controlled air pump^[^ ^50^ ^]^
Milk Remove/Intake	Monitoring milk removal by weighing the mother^[^ ^51^ ^]^
	A mechanical device for measuring infant sucking behavior^[^ ^52^ ^]^
	Infant test weighing indicating milk consumption^[^ ^53^ ^]^
Experiment/Simulation	Tekscan pressure sensor strip on mothers^[^ ^54^ ^]^
	The breast model of milk extraction during breast‐feeding^[^ ^43^, ^55^ ^]^
	Bio‐inspired breastfeeding simulator^[^ ^56^ ^]^

### 2.1 Ultrasound Imaging

In the late 1970s, US imaging was recognized as a noninvasive and radiation‐free technique to investigate latching dynamics during breastfeeding.^[^ ^22^, ^24^ ^]^ As imaging resolution of US improved, greater knowledge could be obtained of the tongue's movement, intra‐oral chamber geometry, and nipple position during sucking.^[^ ^57^ ^]^ US imaging has been employed, both during breastfeeding and bottle‐feeding, with attempts to elucidate the mechanism by which the infant removes milk from the breast. However, variations in scanning techniques, equipment, and imaging planes have given rise to conflicting reports in this field.^[^ ^58^ ^]^

Advances in US imaging highlighted the anatomical differences between the lactating and non‐lactating breast.^[^ ^59^ ^]^ These discoveries unveiled the importance of nipple position in the infant's mouth in relation to the suck/swallow/breathe reflex.^[^ ^60^ ^]^ Geddes et al. employed an US technique to study the movement and position of infants’ tongue and palate during breastfeeding. Through the processing of US images, nipple diameter, tongue distance, and nipple hard soft palate junction distance were all measured during a sucking cycle. The sucking cycle begins when the vacuum is set to a baseline level, increases with the lowering of the tongue, and reaches a maximum when the tongue is at its lowest position. While the tongue lowers, milk begins to flow. Afterward, the tongue then rises until coming to a rest, when the milk flow stops. Also, intra‐oral vacuums were monitored concurrently through a milk‐filled tube connected to a pressure transducer. The US imaging exhibited that the descending movement of the posterior tongue led to an increase in an intra‐oral vacuum and milk flow from the nipple into the infant's oral cavity; the peak vacuum was achieved when the tongue was in its lowest position. The study elucidated the major role of vacuum in milk removal and the significance of tongue mobility.^[^ ^61^ ^]^ Few contemporary studies^[^ ^43^, ^62^ ^]^ also conducted ultrasound measurements along the longitudinal plane of the nipple in the tongue‐up and tongue‐down positions. They measured the distance that the nipple tip moved to the hard‐soft palate junction, as well as, the distance that the mid‐tongue lowered. They reported the importance of tongue movement (tongue lowered) for milk flow. Yet, the role of vacuum is still a matter of controversy in terms of milk removal, particularly as the increased vacuum has not been shown to increase the volume of milk removed.^[^ ^63^ ^]^

Alatalo et al., using the same ultrasound device settings and a similar intra‐oral vacuum setup as outlined in the previous reference, studied the dimensional changes of the nipple inside infants’ oral‐cavity throughout breastfeeding. They additionally used two pressure mapping sensors below the maxilla and the mandible to capture peripheral oral pressure applied by the infant oral cavity on the areola during breastfeeding. Using MATLAB, the average dimensions of the nipple's width and length were characterized during the tongue's movement cycle. Their findings indicated that there is an oscillatory positive pressure profile on the breast from both the maxilla and mandible, differing from previous clinical data showing that only the mandible of an infant moves during breastfeeding. Furthermore, the calculated strain ratio of the nipple during latching in this study implies the significance of strain value difference between lactating and non‐lactating breasts. This suggests that assumptions and parameters being used for non‐lactating breasts cannot explicitly apply to lactating breasts.^[^ ^42^ ^]^

To date, US imaging has been the most commonly practiced technique and the initial modality of choice for the evaluation of breastfeeding mechanics. Although safe and relatively inexpensive, US utilization for this purpose requires a sound understanding of both infant and breast anatomy and the ability to implement dynamic scanning techniques unique to this application. Furthermore, all features of the dynamic breastfeeding process may not be obtained via US imaging alone. The measurement of intra‐oral vacuum or application of biomechanical forces is absent in this technique, and both have a significant role in proper latching and milk extraction. These particular shortcomings are better addressed by multimodal sensing, which will be discussed in the following sections.

### 2.2 Intra‐Oral Vacuum Measurements

Intra‐oral vacuum has been recognized as a key role in sucking patterns and milk extraction during breastfeeding. One prevailing theory suggests that negative pressure (i.e., intra‐oral vacuum) created by the infant is the primary mechanism of milk removal.^[^ ^30^, ^64^, ^65^, ^66^ ^]^ The development of devices to record pressure measurements directly inside an infant's mouth led to the comprehensive capture of sucking microstructure and encouraged several research groups to examine intra‐oral vacuum during bottle‐feeding through a customized feeding bottle with an embedded pressure sensor.^[^ ^67^, ^68^, ^69^, ^70^ ^]^ Additionally, other experimental studies have monitored variations in intra‐oral vacuum during breastfeeding using a pressure sensitive fluid‐filled catheter, which was situated adjacent to the mother's nipple.^[^ ^44^, ^71^, ^72^ ^]^

Sucking microstructure, incorporating the number of sucks, sucks per burst, number of bursts, intra suck interval, and maximal sucking pressure can be acquired through intra‐oral vacuum measurement, and it provides information about the feeding ability of infant throughout latching and feeding. Chen et al.^[^ ^46^ ^]^ proposed a device with an air‐based pressure transducer, providing an objective assessment of infants’ sucking microstructure. Utilizing this device, they recorded the intra‐oral vacuum and compared the sucking microstructure for both breastfeeding and bottle‐feeding. Their study found a measurable difference in the intra‐suck interval between breastfeeding and bottle‐feeding, and no difference for other microstructure parameters. Additionally, they confirmed the feasibility of using an air‐based pressure transducer for intra‐oral vacuum measurement as an alternative to other apparatus which utilize a fluid‐filled feeding tube. Geddes et al.^[^ ^48^ ^]^ employed a silicone tube filled with sterile water, situated adjacent to the mother's nipple, which was connected to the pressure transducer. In this study, intra‐oral vacuum measurements were synchronized with US imaging to study the tongue position with regard to the vacuum profile and the amount of milk in the infants’ oral cavity. In another work, Geddes et al.^[^ ^44^ ^]^ developed a phantom teat, which comprised a hollow silicone tube, a membrane, and a duck valve to measure intra‐oral vacuum during feeding. The investigation found that milk removal from an experimental teat solely depends on the exerted vacuum caused by the infant's tongue movement.

In the year 2020, Jiang et al.^[^ ^56^ ^]^ introduced a bio‐inspired breastfeeding simulator. This experimental apparatus mimics infant oral behavior and milk extraction, applying the breastfeeding mechanism in vitro. The development of the device arose from the sets of clinical data comprising measurements of natural intra‐oral vacuum, the pressure from the infant's jaw, tongue, upper palate, and nipple deformation on the breast areola area. The development of such a breastfeeding simulator has opened new avenues for further understanding the bio‐mechanics of breastfeeding and formulates a foundation for future breastfeeding device development.

### 2.3 Milk Removal

Successful breastfeeding requires the infant to remove milk efficiently from the breast. One of the most common reasons for mothers to give up on breastfeeding is a perception of insufficient milk intake.^[^ ^73^, ^74^ ^]^ There have been several experimental and computational studies to better understand the dynamics involved with milk transfer during breastfeeding. Infant test weighing is the most commonly used technique for measuring milk intake during breastfeeding episodes. In this method, the infants are weighed before and after feeding; the weight gain indicates the total milk consumption throughout feeding.^[^ ^75^, ^76^ ^]^ Often a scale‐based technology is used as a gold standard to cross‐validate other scoring systems in estimating milk removal. In the year 2020, Perrella et al.^[^ ^77^ ^]^ conducted pre/post‐feed weight measurement using electronic scales and the preterm breastfeeding assessment tool (PBAT) scores for 60 preterm infants born less than 33/40 and 33 to 39/40 postmenstrual age. The study reported that in comparison with test weighing using a scale (e.g., ±2 g), PBAT is prone to subjective errors of 26% and 47%, when particularly in estimating the transfer of half of or the fully prescribed volumes, accuracy 26% and 47%, respectively. In another study, kent et al.^[^ ^78^ ^]^ leveraged test weighing (scale‐based technology) to assess milk production's impact on breastfeeding confidence in mothers of full term infants.

Kron et al.^[^ ^52^ ^]^ developed an apparatus for recording the baseline intra‐oral vacuum and volumetric flow of sucking, as a function of time during breastfeeding. In this study, a test nipple was designed so that it was impossible for infants to initiate milk flow with mouthing or chewing movements, rather permitting only a sub‐atmospheric intra‐oral vacuum developed by infants for milk extraction. The outputs from the apparatus were the volumetric flow from a burette and pressure‐time curves from a transducer. Through hydrodynamic analysis, the efficiency of sucking by the infant was measured. The efficiency of sucking may be defined as the relation between the rate at which energy is used to generate intra‐oral pressure and the resultant rate of nutrient flow.^[^ ^52^ ^]^ Although this study did not explicitly investigate if infants required more than just suction to be fed well, the concept of the apparatus and the method of sucking measurements opened the way to study the primary mechanism driving milk removal. Yet, proper breastfeeding involves both latching and sucking and, ultimately, the entire dynamic process needs to be examined.

Moreover, several research groups quantified infants’ milk intake by injecting deuterium oxide into mothers, and then analyzed saliva samples collected from both the mother and baby. Estimations of milk intake can be made from the amount of deuterium found in the infant's body, as the deuterium comes only from maternal milk and correlates to the amount of milk consumed.^[^ ^79^, ^80^ ^]^ Some studies reported the success of the deuterium oxide dose‐to‐the‐mother technique, particularly among breastfed infants less than 6 months old.^[^ ^80^, ^81^ ^]^ However, after 6 months, the amount of infant milk intake is too variable for this to be a useful tool. Furthermore, comparing the studies which employed the dose‐to‐the‐mother technique indicates that the technique cannot be sufficiently reliable since there are discrepancies in the amount transferred based on the size, level of activity, and nutritional status of the mother.^[^ ^82^, ^83^ ^]^ It is worth noting that for the deuterium oxide dose‐to‐the‐mother technique to be considered clinically safe, the enrichment level should stay less than 0.1% in the mothers’ bodies.^[^ ^76^ ^]^

### 2.4 Computational, Statistical, and Mathematical Modeling

Computational modeling of biological systems including human lungs,^[^ ^84^, ^85^ ^]^ brain,^[^ ^86^, ^87^ ^]^ blood vessels,^[^ ^88^, ^89^ ^]^ and heart,^[^ ^90^ ^]^ is gaining momentum as a diagnostic tool. Beyond their initial applications, these novel techniques have been applied to breastfeeding in an effort to better understand the underlying mechanics. Elad et al.^[^ ^43^ ^]^ presented a 3D model of the breast and lactiferous tubes to mimic dynamic characteristics during breastfeeding. Their model demonstrated that milk extraction is caused by fluctuating sub‐atmospheric pressures within the infant's oral cavity during latching and milk extraction and not by mouthing of the nipple‐areola complex. The anterior tongue was noted to sit between the nipple‐areola complex and the mandible, and functioned as a rigid body with the cyclic movement of the mandible. The posterior tongue did move in a peristaltic wave to aid in swallowing, but this did not compress the breast. Azarnoosh et al. used fluid‐structure interaction (FSI) simulations to study the two existing theories in expressing milk from a nipple; FSI is the multiphysics study on how deformable structures interact with an internal or surrounding fluid flow. The simulated results were cross‐validated with clinical US images. This study concluded that the intra‐oral vacuum indeed plays a primary role in milk removal from the nipple, and although the contribution of the jaw movement is not notable to milk removal rate, it may facilitate the expression of milk by deforming the areola in a manner that lowers the shear stress inside the main duct.^[^ ^55^ ^]^

Zoppou et al.^[^ ^91^ ^]^ employed a mathematical model based on a quasi‐linear poroelastic theory to analyze milk extraction between infant breastfeeding and the use of a breast pump. Although the results of this theoretical model were not conclusive, they indicated that throughout suckling, both the peristaltic motion of the tongue (the stripping action) and the negative suction pressure are equally important. Finally, computational modelling has been used to examine the health impact of breastfeeding. Bartick et al. performed Monte Carlo simulations modeling a hypothetical cohort of U.S. women from age 15 to 70 years, and their children's health outcomes from birth to 20 years of age. They demonstrated that suboptimal breastfeeding in the United States was correlated with an excess of 3340 premature maternal and child deaths (95%), due to seven different diseases.^[^ ^92^ ^]^ Thus, computational, statistical and mathematical modelling have proven useful in the study of breastfeeding from the finest intricate details of latching to larger public health implications.

While intra‐oral pressure plays a role in feeding mechanics, for simulation modeling to be a true representative of a breastfeeding mechanism, the simulation needs to incorporate the maternal breast's dynamic action in relation to oxytocin release from myoepithelial contraction of the ductal tissue. For example, Quezada et al.^[^ ^93^ ^]^ reported a multi‐layer transport modeling to analyze the toxins present in breast milk. In this work, the transport of three toxins moving from the bloodstream into the mammary glands “ducts was investigated. However, the application of their simulation was to shed light on toxin transport as a pre‐disposing factor to breast cancer. Thus, the inclusion of physiological parameters and the mammary glands” transport phenomena is an integral part of breastfeeding simulation.^[^ ^94^, ^95^ ^]^

## 3 Discussion

Despite years of research, we still lack effective and reproducible methods to measure infant nursing ability and latching dynamics. Thus, there are significant difficulties in diagnosing and treating issues related to nursing difficulty. The importance of measuring milk intake must not be trivialized; breastfeeding is a dynamic process that involves both latching and sucking, and efficient milk transfer is vital to successful lactation. Often it is not clear whether insufficient milk transfer is related to the feeding mechanics of the infant, maternal factors, or a unique issue affecting the breastfeeding dyad.

Insufficient milk transfer can be a consequence of low maternal milk production, localized milk stasis, and/or blocked/plugged ducts.^[^ ^96^ ^]^ In addition, inadequate milk production can be induced if the breasts are not drained sufficiently and frequently enough due to poor latching, poor breastfeeding technique, and/or illness of the infant.^[^ ^97^ ^]^ Beyond successful latching, mothers’ early breastfeeding cessation is another barrier that needs to be overcome to promote breastfeeding's role in health and illness prevention throughout people's full lifespan. More than 60% of mothers in the US reported the cessation of breastfeeding in the first month, mostly attributed to difficulty with suckling and latching.^[^ ^98^, ^99^ ^]^ Maternal nipple pain, painful breasts, insufficient milk intake, poor latch, and suboptimal weight gain are reported as the most common reasons for breastfeeding cessation. Frequency and sufficiency of milk extraction are the most crucial factors in regard to successful breastfeeding.^[^ ^100^ ^]^ During the in‐hospital perinatal period, test‐weighing is often used to measure the volume of intake during breastfeeding. Once discharged, mothers can only try to utilize clinical cues, such as audible swallowing, sucking patterns, and predictions of infant satiety to determine milk intake. However, the precision of these cues with regard to the volume of milk intake has never been examined. To this end, surveyed mothers have reported that they lack an effective and objective measurement of milk intake and that they were unable to accurately use these subjective cues during the first four weeks post‐discharge to determine adequate milk intake.^[^ ^101^ ^]^

In hopes to reconcile the paucity in understanding, advanced techniques utilizing US and finite element modeling have been developed. US imaging is the most commonly practiced technique and the primary modality of choice for evaluating latching dynamics. However, all features of the dynamic breastfeeding process may not be obtained via US imaging alone and demand a synchronization of other sensing modalities such as vacuum measurement of the intra‐oral cavity,^[^ ^45^, ^46^ ^]^ pressure sensor strip, or the commercialized NTrainer system.^[^ ^50^ ^]^ However, these systems only provide limited data, as there only single sensors and no full understanding of force distribution to understand latching dynamics between the infant and breast. In the framework of measuring milk intake during breastfeeding, weighing (scale‐based technology) is the most commonly used technique. In reference to computational modeling and simulation, unquestionably, they have paved the way toward a better understanding of underlying latching dynamic mechanisms. Yet, they are limited to capture the maternal breast's dynamic action. Despite the aforementioned studies and variety of employed techniques, there remains a scarcity of objective data regarding breastfeeding by means of a quantitative device, which permits a convenient and real‐time objective measurement of infants’ latch and milk transfer in both the home and clinical settings.

Despite all available advances for the prevention and treatment of breastfeeding difficulties, several aspects of latching performance demand a deeper understanding and consensus on management strategies. There is an urgent need for a quantitative assessment of infant latching and feeding dynamics through prospective controlled studies, addressing the diagnosis and efficacy of treatment concerning breastfeeding difficulties.

## 4 Conclusion

Since the early efforts to characterize the behaviors of infants feeding movement in the 1950s, there has been a continued development of instruments, techniques, and computational studies to further the understanding of breastfeeding dynamics. It is clear that the developed instruments and techniques have shed light upon breast anatomy and given us an improved understanding of infants’ sucking microstructure. Despite these findings, there is still much controversy in the medical and lactation professions regarding the classification, consequence, and treatment outcomes of various breastfeeding difficulties and disorders. This lack of agreement necessitates an urgent need for quantification of latching dynamics in conjunction with milk removal rate through prospective clinical studies. Once we have more clarity into the world of infant latching and feeding, only then can we provide the best care possible to our most vulnerable patients.

## Conflict of Interest

The authors declare no conflict of interest.

## Author Contributions

B.M. and S.D.R. conceived of the presented review paper. M.T. took the lead in writing the manuscript with support from B.M. and S.D.R. All authors edited and formatted the final draft.
